# Lipid droplets: a cellular organelle vital in cancer cells

**DOI:** 10.1038/s41420-023-01493-z

**Published:** 2023-07-20

**Authors:** Yi Jin, Yanjie Tan, Jian Wu, Zhuqing Ren

**Affiliations:** 1 Key Laboratory of Agriculture Animal Genetics, Breeding and Reproduction of the Ministry of Education & Key Laboratory of Swine Genetics and Breeding of the Ministry of Agriculture and Rural Affairs, College of Animal Science, Huazhong Agricultural University, Wuhan, 430070 Hubei P. R. China; 2 Institute of Biomedical Sciences, Key Laboratory of Animal Resistance Biology of Shandong Province, College of Life Sciences, Shandong Normal University, Jinan, 250014 Shandong P. R. China; 3 Hubei Hongshan Laboratory, Wuhan, P. R. China

**Keywords:** Organelles, Cell biology

## Abstract

Lipid droplets (LDs) are cellular organelles comprising a core of neutral lipids (glycerides, sterols) encased within a single phospholipid membrane, responsible for storing surplus lipids and furnishing cellular energy. LDs engage in lipid synthesis, catabolism, and transport processes by interacting with other organelles (e.g., endoplasmic reticulum, mitochondria), and they play critical roles in regulating cellular stress and immunity. Recent research has uncovered that an elevated number of LDs is a hallmark of cancer cells, attributable to their enhanced lipid uptake and synthesis capacity, with lipids stored as LDs. Depletion of LDs in cancer cells induces apoptosis, prompting the emergence of small molecule antitumor drugs targeting LDs or key factors (e.g., FASN, SCD1) within the lipid synthesis pathway. Advancements in LD isolation and artificial synthesis have demonstrated their potential applicability in antitumor research. LDs extracted from murine adipose tissue and incubated with lipophilic antitumor drugs yield drug-coated LDs, which promote apoptosis in cancer cells. Furthermore, LDs have been employed as biological lenses to augment the resolution of subcellular structures (microfilaments, microtubules), facilitating the observation of intricate structures within thicker cells, including cancer cells. This review delineates the functional and metabolic mechanisms of LDs in cancer cells and encapsulates recent progress in LD-centered antitumor research, offering novel insights for tumor diagnosis and treatment.

## Facts

- Lipid droplets (LDs) serve as vital reservoirs for neutral lipids, playing a pivotal role in maintaining cellular energy balance, lipid homeostasis, and signaling.
- In contrast to normal cells, cancer cells exhibit heightened metabolic activity, showcasing amplified lipid uptake and synthesis.
- Cancer cells possess a notable characteristic of having a significant quantity of cellular LDs.
- Targeting LDs provides a huge research prospect for tumor therapy.

## Open questions

- What are the potential functions of LDs in cancer cells?
- How can we utilize the biological characteristics and functions of lipid droplets to inhibit cancer cell growth and treat tumors?
- Whether drugs targeting LDs can be a way to treat tumors?

## Introduction

In 1674, Levenhoek discovered many oil droplets (now commonly called lipid droplets, LDs) in milk with a microscope, which was the first membranous organelle discovered by humans [1]. The essential difference between LDs and other membranous organelles is that LDs are enveloped by a single phospholipid membrane with neutral lipids inside, including glycerides, cholesterol and sterols. LDs are ubiquitous in cells from archaea to eukaryotes and are the earliest membrane organelles to evolve [2]. LDs emerged in the sense of allowing cells to fix energy in the form of fats when the energy supply is rich and to break down these stored fats to sustain cellular life activities when the energy supply is low [3, 4]. With advances in LDs isolation and bioanalysis, researchers have analyzed the biological composition of LDs and found that the surface of LDs is surrounded by many proteins (called LD proteins) that have numerous origins [5], some belonging to the LD itself and involved in the regulation of LDs structure and function, and others originating from the cytoplasm or other organelles, which confer multi-functions to the LD besides the storage of excess lipids [6].

A distinctive feature of cancer cells is the large number of cellular LDs. Cancer cells are metabolically active compared to normal cells, with greatly enhanced lipid uptake and synthesis. Cancer cells have elevated expression levels of the fatty acid transport protein such as FABPs, which contributes the cells to take up large amounts of external fatty acids. In addition, the expression levels of the fatty acid synthase (FASN) are elevated and the de novo synthesis pathway of fatty acids is activated, synthesizing large amounts of fatty acids. These fatty acids are catalyzed by acyltransferases to synthesize glycerides, which are stored in LDs [7–9]. The larger number and diameter of LDs, the more fats are stored [10]. LDs in cancer cells can provide energy and essential lipid precursors for rapid cancer cell proliferation [11, 12]. In addition, since LDs are also phospholipid reservoirs of cells, they can provide phospholipid membranes for the synthesis of membranous organelles in cancer cells to accommodate the rapid growth of cancer cells [13]. Studies blocked LD formation by inhibiting lipid synthesis pathways such as FASN and found that cancer cells lacking LDs were more prone to apoptosis. Further studies found that LDs, in addition to providing energy to cancer cells, also contribute to remove reactive oxygen species (ROS) from cells and reduce cellular stress [14, 15]. Therefore, LDs are important for the survival of cancer cells.

Several antitumor drugs (e.g., sorafenib) are highly lipophilic and not easily absorbed and metabolized by the body, and even through liposome encapsulation still cannot effectively enhance the utilization of the drug. Recently, researchers extracted LDs from adipose tissue and encapsulated lipophilic drugs into LDs and co-incubated these LDs with cancer cells. It was found that cancer cells could take in these LDs into the cells, and as the LDs were catabolized, the drug entered the cells and subsequently acted to kill the cancer cells. This discovery opens up the possibility of using LDs as a biological agent in the field of anticancer. In addition, in the field of cancer cell research, LDs are used as biological lenses to increase the resolution of microscopic observation of subcellular structures and contribute to the resolution of cellular metabolic mechanisms.

This review illustrates the important role of LDs as an organelle in regulating the proliferation and growth of cancer cells. It also summarizes the potential applications of LDs as a nano-biologic agent in the recent anticancer research field. This review provides new insights into the diagnosis and treatment of tumors.

### LDs are center of lipid metabolism in cancer cells

During tumorigenesis, cancer cells acquire various metabolic alterations to overcome the metabolic challenges of rapid proliferation and survival under unfavorable conditions. Changes in cancer metabolism are driven by the activation of oncogenes or loss of tumor suppressor genes and alterations in cellular signaling [16–18]. Recent evidence suggests that reprogramming of lipid metabolism, including changes in neo-lipogenesis, FA oxidation, and phospholipid and neutral lipid metabolism, is critical for various aspects of tumorigenesis [19, 20]. Although most normal cells preferentially use extracellular lipids to synthesize new structural lipids, cancer cells can enhance new FA synthesis to meet their lipid requirements or maintain proliferation in a lipid-poor microenvironment, regardless of the level of extracellular lipids [20, 21]. Upregulated SREBP signaling contributes to increased synthesis of phospholipids, TAG and cholesterol and promotes cell survival and tumor growth [22, 23].

From an evolutionary perspective, organisms began to use triglycerides to store energy, leading to the creation of LDs as organelles, which allowed cells to no longer be adversely affected by energy shortages and greatly facilitated their survival in materially deficient environments [24]. However, recent lipidomic studies have revealed that LDs store many lipid precursors, including vitamin E, vitamin A, retinyl esters, and Retinol [25, 26], besides triglycerides. These lipids are essential for antioxidant and hepatic metabolism, and some hormone secretion and a deficiency of LDs can lead to secondary storage of the relevant lipid precursors and cause disease. In addition, LDs contain many lipids signaling molecules, including steroid hormones and FA signaling, which play essential roles in cellular signaling [27]. In mitochondrial biosynthesis, for example, LDs are an essential source of cholesterol [2, 28]. In addition, precursors of eicosanoids stored in LDs, including prostaglandins, thromboxanes, and leukotrienes, are essential for cellular immunity [29, 30], and inhibition of LD function results in defective cellular immunity [31].

In addition to the primary function of storing lipids, LDs are also deeply involved in regulating lipid synthesis and metabolism. Cells can synthesize neutral lipids de novo from fatty acids, which requires the catalysis of glycerol-3-phosphate acyltransferase (GPAT) and diacylglycerol acyltransferase (DGAT) [32, 33]. When LDs are generated on the endoplasmic reticulum surface, these two proteins are transferred to the LD surface with the flow of phospholipids. Together with their hydrophobic helix structure, these proteins can firmly bind to LDs. Therefore, free LDs in the cytoplasm still have a strong capacity for glycerol ester synthesis, which significantly contributes to cell lipid synthesis and storage capacity [34]. In addition to lipid synthesis-related enzymes, there is also a class of lipases on the LD surface, including ATGL, HSL, and MGLL, which are responsible for catalyzing the hydrolysis of triglycerides, diglycerides, and monoglycerides, respectively [29, 30]. The protein structures of these three enzymes are hydrophobic and can be stably localized on the LD surface.

**Fig. 1:**
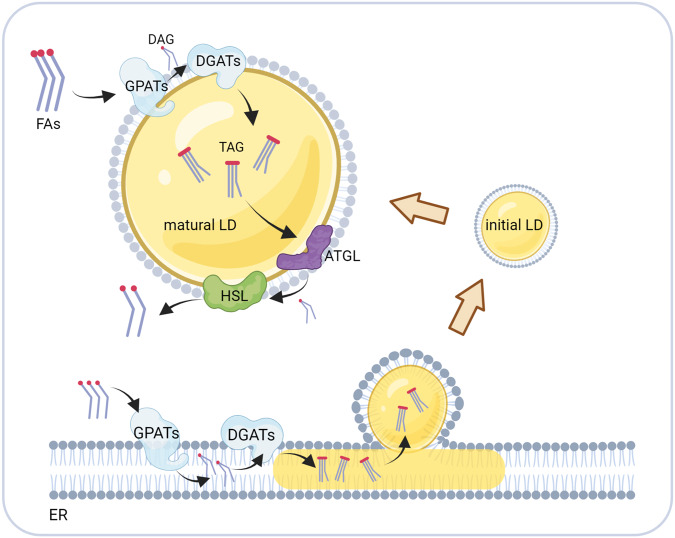
LDs are the center of cellular lipid metabolism. GPATs and DGATs localized at the endoplasmic reticulum (ER) synthesize fatty acids into glycerides, which are stored in the intercalated layers of the ER phospholipid membrane and eventually form LDs in a budding manner. Initial lipid droplets (iLDs) form mature lipid droplets after the recruitment of proteins. Mature LDs contain glycerol ester synthase and lipase at the surface, which are involved in lipid synthesis and catabolism.

Moreover, the activities of these enzymes are regulated by CGI-58, PLIN1, and PKC to ensure cellular energy supply. Therefore, LDs provide sites for lipid storage, synthesis, and degradation. LDs also regulate cellular fatty acid flow, balance cellular energy storage, and release, maintain cellular lipid homeostasis, and are the centers of cellular lipid metabolism (Fig. 1), which is essential for cell survival [35, 36].

Recent studies have identified many contact sites between LDs and other organelles and have observed closer-than-expected interactions between LDs and other organelles [37, 38]. In addition, mutated disease factors in many genetic diseases are contact site proteins between LDs and other organelles, and LD–organelle contacts play essential roles in the onset and progression of many diseases [39, 40].

It has become a central research focus to study how organelles communicate to achieve coordinated behavior within a cell. The exchange of materials and information between organelles is carried out by diffusion, cytoskeleton-based directed transport mechanisms, vesicle trafficking, and organelle contact points. Physical connections between organelle surfaces enable direct communication at contact points [41–43]. LD–endoplasmic reticulum contacts were extensively studied first in the literature. Recently, proteins such as Seipin, SNX14, SCAR20, Mdm1, VPS13, and others were found to be localized at the interface of LD–endoplasmic contacts and are involved in LD–endoplasmic contacts [42, 44–48]. Many LD contact point mutations are linked to neurodevelopmental and neurodegenerative diseases and metabolic disorders [48, 49]. In addition, the contact between LDs and the mitochondria is regulated by proteins such as PLIN5, PLIN1, and Mnf2 [50–54]. There are two scenarios of LD–mitochondria interactions. First, LD–mitochondria contacts promote the oxidative decomposition of LDs and provide large amounts of energy quickly and efficiently. Second, mitochondria wrap around LDs (periplasmic LD mitochondria), providing ATP for lipid synthesis, promoting lipid accumulation in LDs, and encouraging increased LD volume [50, 55].

### LDs contribute to alleviate cellular stress in cancer cells

Cancer cells display elevated rates of ROS production due to oncogenic mutations, loss of tumor suppressors, accelerated metabolism required to sustain rapid growth, and adaptation to various stresses imposed by the tumor microenvironment (i.e., nutrient and oxygen deprivation, infiltrating immune cells) [56, 57]. Thus, cancer cells may experience oxidative stress both during oncogene-driven nutrient overload and rapid metabolism, as well as during nutrient and/or oxygen deprivation in poorly vascularized tumors [58]. Prolonged exposure to high levels of ROS may lead to oxidative stress-dependent cell death, with cancer cells simultaneously upregulating antioxidant pathways to maintain redox homeostasis. Hypoxic cancer cells may upregulate FA uptake to compensate for impaired de novo lipogenesis [59, 60]. Furthermore, hypoxia inhibits FA oxidation and can induce the formation of LDs as temporary stores of energy for use upon reoxygenation [60, 61].

**Fig. 2:**
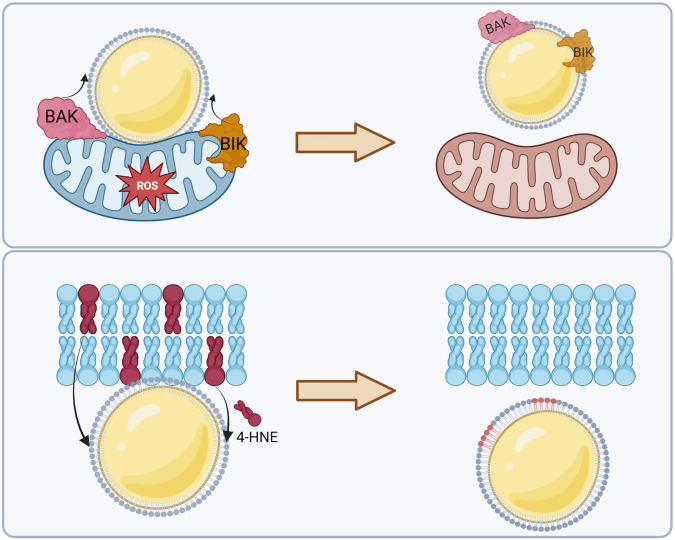
LDs are involved in the removal of harmful proteins and lipids. LDs can contact mitochondria and transfer harmful proteins BAK and BIK, which cause elevated ROS on the mitochondrial surface, to their surface, thus reducing mitochondrial damage. In addition, LDs can transfer oxidized phospholipids (4-HNE) from the cell membrane to their surface, thus alleviating phospholipid membrane damage.

The accumulation of metabolic waste will stimulate the cellular stress response and lead to apoptosis in severe cases [62]. In recent years, studies related to the anti-stress functions of LDs have been published in *Cell* and other top journals worldwide, which has led to the recognition that LDs have essential research value in the field of cellular stress [14, 15, 63]. The anti-stress function of LDs is mainly derived from their buffering effect. First, LDs can absorb excess fatty acids and avoid lipotoxicity. Cells in an environment with high fatty acid content (e.g., small intestinal epithelial cells that absorb nutrients from food or hepatocytes that develop fatty liver) form a large number of LDs that esterify fatty acids to triglycerides, thereby reducing fatty acid toxicity and protecting cells [14, 64, 65]. In addition, LDs can temporarily absorb and store fatty acids, rapidly reducing fatty acid levels and preventing lipotoxicity. Second, LDs in nerve cells can exchange phospholipids with the cell membrane, thus transferring peroxidized phospholipids [66, 67] from the cell membrane to itself and typical phospholipids to the cell membrane, after which the LDs can be phagocytosed and broken down by autophagic lysosomes, completing the recycling process of phospholipids [68–70]. For example, LDs are involved in clearing oxidative phospholipids of a membrane in the brain. 4-Hydroxynonenal (4-HNE) is an endogenous lipid peroxidation product derived from the lipid peroxidation of ω-6 polyunsaturated fatty acids (PUFAs) in tissues and cells. LDs in contact with the membrane can transfer 4-HNE from the membrane to its surface, thus reducing oxidative damage to the membrane (Fig. 2). This process is critical for stress resistance in the brain and indicates that LDs in nerve cells may play a protective role and thus could be a therapeutic target for diseases such as neurodegenerative disorders. Finally, LDs can transfer oxidatively damaged proteins (including some unfolded proteins, BAK, BAX, etc.) from the endoplasmic reticulum and mitochondrial surface to themselves, thus inhibiting endoplasmic reticulum stress, as well as oxidative damage to the mitochondria, and reducing the occurrence of apoptosis [71].

The above findings suggest that LDs may play the role of cleaners in cells, absorbing excess fatty acids, peroxidized phospholipids, and oxidatively damaged proteins, thereby reducing the damage of these harmful substances to cells. Furthermore, after completing their mission, LDs are broken down by autophagic lysosomes, thus completing the recycling of substances. This mechanism dramatically enhances cell survival, especially for highly specialized cells such as nerve cells, where mitigating oxidative stress can prevent nerve cell death and is of great value to human health.

### LDs can be used for anticancer drugs delivery and cancer cells killing

The neutral lipid cores of LDs are capable of solubilizing numerous lipophilic substances, including vitamins A and E. Recent research has discovered that LDs serve as sites for drug accumulation and metabolism. Lasonolide A (LasA), a macrolide isolated from marine sponges, exhibits cytotoxicity against specific cancer cell types at nanomolar concentrations [72]. The enzyme LDAH, which encodes the metabolite serine and belongs to the hydrolase family, is localized on LD surfaces and is instrumental in LasA’s action by metabolizing it to produce toxicity. LasA, a lipid-soluble drug, predominantly localizes within LDs, whereas LDAH is found on LD surfaces and in the endoplasmic reticulum [73]. It is postulated that LDAH emerges from the ER concomitant with LD formation from the cytoplasmic leaflets of the ER, subsequently migrating to the LD surface [2, 73]. Consequently, LD cleavage may transpire at the membrane interface, either at the ER membrane or on the LD surface. Augmenting cellular LD content through treatment with oleic acid, DGAT inhibitors (T-863 and PF06424439), or long-chain fatty acid acyl-CoA synthase inhibitors (Triacsin C) diminished cellular LD content. The IC50 of LasA correlates with LD content in cellular membranes, as indicated by measurements of LasA efficacy. Elevating LD content with oleic acid enhanced drug potency, while impeding triglyceride and cholesteryl ester synthesis reduced drug potency [74].

A recent scientific investigation revealed that LDs form a metastable reservoir, bolstering resistance against Mycobacterium tuberculosis [75]. During antimicrobial therapy, robust lipid binding facilitates drug transfer from host organelles to intracellular targets. Bedaquiline (BDQ), characterized by its lipophilicity, is an exemplary antituberculosis drug [76, 77], exhibiting the capacity to permeate tissue while circumventing nonspecific binding and host sequestration [78–80]. Previous studies have documented the interaction between *Mycobacterium tuberculosis* (Mtb) and host LDs [81], demonstrating a high enrichment of antibiotics within LDs. Transmission electron microscopy unveiled extensive physical contact between LDs and Mtb, as well as tightly enclosed vacuoles containing Mtb. The LD surface protein Perilipin 2, which associates with *M. marinum* intracellularly, has also been identified [82]. These diverse interaction levels may contribute to the heterogeneity of BDQ accumulation. Since Mtb secretes lipase [83, 84], the close proximity between LDs and Mtb leads to the degradation of LD-bound BDQ, facilitating its transfer from the host reservoir to Mtb. Co-treatment with pralidoxime, which diminishes LD levels, significantly reduced BDQ abundance in Mtb and hindered preloading. Consequently, BDQ transfer between bacteria and LDs is plausible. Nonetheless, LDs are not always necessary for BDQ transfer between bacteria and Mtb. The induction of LD formation by the addition of oleic acid did not prevent BDQ transfer. Thus, LDs may function as an accessible reservoir rather than a sequestering agent for BDQ.

As mentioned above, LDs can be used as bio-nanomaterials for anticancer drug delivery, but as an exogenous macromolecular substance, how drug-encapsulated LDs enter cancer cells remains an important scientific question. Several biological processes are involved in targeting the entry of macromolecules into cells, and endocytosis is the most likely avenue [85, 86]. There are various types of endocytosis, including lectin-mediated endocytosis, micropinocytosis, caveolin-mediated endocytosis, and lipid-raft-mediated endocytosis [85, 87]. Through targeted inhibition of the critical proteins in different endocytosis processes, it was found that only lectin-mediated endocytosis and micropinocytosis affected the uptake of LDs via cancer cells [87]. In particular, lectin-mediated endocytosis largely determined the number of LDs entering cancer cells [87]. The process of lectin-mediated endocytosis is as follows. First, lectins aggregate in the cell membrane to form a region of fixed curvature that continues to involute over time, eventually reaching an utterly spherical shape [88]. Next, actin filaments nucleate around the base of the invagination by the Arp2/3 complex; actin continuously polymerizes and pulls the lectin-coated membrane coupled to it toward the cell interior; and, finally, Amphiphysin, Dynamin, and Endophilin participate in the break of the closure to form phagocytic vesicles [89]. The LDs are carried into the cell via the phagocytic vesicles at the intracellular traps and subsequently bind to lysosomes [89]. The phagocytic vesicles open, and the LDs enter the cytoplasm to perform their functions. However, many questions remain unclear about this process, such as how LDs trigger endocytosis at the cell surface, the relevant recognition receptors, and whether LDs entering the cell are rapidly broken down by lysosomes. The answers to these questions require further in-depth analysis.

In the cell, organelles are responsible for many life-sustaining processes, including DNA replication, cellular metabolism, and protein synthesis [74]. The small size and unique function of organelles make them suitable candidates for enriching the library of biomaterials for the proper treatment of diseases [90]. LDs, an organelle type abundantly present in adipocytes at the nano- and microscopic scales, are well-suited for use as bio-nanomaterials in cancer therapy applications due to their unique structures and critical roles in lipid metabolism and thermogenesis. In addition, a large quantity and ease of separation of LDs from other cellular components may facilitate large-scale production of LDs.

As a result of their lipid-rich compartments, LDs are excellent containers for lipophilic drugs and can be used to improve therapeutic outcomes by exploiting the intrinsic link to abnormal lipid metabolism within cancer cells [87]. Photodynamic cancer treatment is enhanced by encapsulating a photosensitizer in LDs, enhancing photodynamic therapy’s efficiency [87]. Furthermore, this cell-derived material is stable in the physiological environment, has controlled physicochemical properties, maintains interactions with other organelles involved in metabolic regulation, and has limited side effects on normal tissues [91].

It was found that LDs play a critical role in facilitating anticancer therapy rather than merely acting as drug carriers. It is possible to manipulate and modulate LDs’ size, size distribution, and intracellular communication, potentially providing a controllable and versatile platform. In addition to maintaining lipid metabolism, LDs also play a crucial role in cellular signaling and inflammation [92, 93]. LD-based systems could be further expanded by combining them with other therapeutic modalities, such as chemotherapy and immunotherapy.

Compared to other drug delivery systems, LDs are stable and biocompatible. It may be possible to avoid the potential side effects of some uncertain components within LDs due to their composition and structure, which are much more complex than those of whole cells. In addition, LDs can be stably preserved after freeze-drying into powder, which may drive further commercialization. Moreover, the surface of LDs can be artificially supplemented with corresponding proteins to enter different cells or interact with specific organelles to improve the targeting of drug delivery. By transforming cell-based therapies into organelle-based ones, new perspectives can be gained on drug delivery, and hope can be gained for clinical application.

### LDs can be used as biocompatible microlenses in cancer cell imaging

Another novel application of LDs was revealed in a recent study. Researchers found that endogenous LDs in mature adipocytes can act as fully biocompatible microlenses to enhance microscopic imaging, with the ability to detect intracellular and extracellular signals [94]. Using LD biolens, enhanced fluorescence images of adenoviruses and lysosomes were obtained. In addition, the study demonstrated that it was possible to reduce the required excitation power by 73% [94]. Optical tweezers were used to finely manipulate the lipids, thereby resolving the target and imaging it in real time inside the cell. Focusing the incident light on LDs made it possible to detect cancer cell fluorescence signals in the extracellular fluid. LDs can be used as biocompatible intracellular microlenses for biosensing, endoscopic imaging, and single-cell diagnostics [94].

The greater thickness of cancer cells compared to some typical epithelial cells makes it more difficult to obtain clear images of the microstructures within cancer cells. Furthermore, optical microscopy imaging techniques are preferred to monitor endoplasmic changes in real time and rapidly detect extracellular signals to obtain a large amount of important information on cellular physiological and pathological processes [95, 96]. It is common to use fluorescent signals during optical microscopy imaging to improve the discrimination of cells and tissues [97]. Typically, the fluorescent signals from subfluorescent signals in cellular structures are fragile, making it challenging to directly use fluorescence methods for intracellular imaging or detection. Increasing the excitation light’s power makes it possible to increase fluorescence intensity, but the risk of phototoxicity to cells increases as well, with a more substantial photobleaching effect in fluorescence intensity [98, 99]. Previous studies used, for example, chloroplasts and erythrocytes as microlenses to achieve amplification for imaging and enhancement in fluorescence signal detection [100].

In comparison to chloroplasts and erythrocytes, LDs are more robust and stable. LDs operate as microlenses in various biological environments and are internalized by various cell types. LDs are also more efficient at magnification and fluorescence enhancement due to their high refractive index and near-spherical shape. Fluorescence imaging becomes possible in contact mode because the extraction efficiency of emitted fluorescence signals is improved, reducing optical power requirements. As a result of the LDs highly convergent excitation light, fluorescence from the extracellular environment surrounding the cell is enhanced in real time. Lensing effects produced by LDs may be applied in miniaturized biosensors, endoscopic analysis, and endoscopic single-cell diagnostics, all of which are fully biocompatible. The use of LDs as intracellular microlenses also offers opportunities for the construction of various endogenous photonic devices.

### Artificial LD technology can solve the problem of LD yield

Since LDs have potential biomedical applications, rapid and extensive quantities of LDs will be needed at a later stage to meet market demands. Cell types other than adipocytes, including cardiomyocytes, monocytes, astrocytes, and epithelia, have been exposed to LDs in several ways. Biochemical mechanisms are mainly responsible for LD accumulation in these non-adipocytes. However, the extraction of LDs from cells is both a costly and complex process, as LDs are difficult to extract, and the appropriate methods are demanding in terms of instrumentation and equipment. Therefore, rapidly acquiring large amounts of LDs via chemical synthesis should be considered for future analyses.

Recent developments in synthetic biology have enabled the synthesis of ATPase-active structures that resemble cells [101]. An LD in vitro system was constructed to study the function of proteins such as microbial LD mini protein (MLDS) and ATGL [102–104]. Moreover, structures similar to LDs were obtained by injecting triglycerides into giant unimolecular vesicles (GUVs), confirming that surface tension affects the directionality of LD growth [105]. Furthermore, microfluidic techniques have been used to investigate the role of COPI in LD protein transport [106]. A recent study identified a facile method for preparing artificial LDs [91]. As a solvent, ethanol was used to dissolve phospholipids and triglycerides, and then the aqueous buffer was added to the organic phase and stirred until the ethanol was evaporated to form a milky white solution. This solution was composed of artificial LDs with uniform particle sizes and was structurally sound [91]. In addition, to achieve better preservation and utilization of LDs, 5% mannitol was used as a lyophilization protectant with a eutectic point of –5 °C. Lyophilized samples were white loose porous powder with a fine, smooth, and uniform surface. With the addition of 2 mL of 0.2% Tween 80 solution, redispersion occurred rapidly, and the powder became white and creamy [91]. Redissolution of the stored samples showed typical morphologies compared to their newly prepared counterparts.

The technology of artificial LDs is rapidly developing. For example, LD preparation methods are becoming more straightforward, decreasing their preparation time. However, the cost of raw materials for LD production in the future must also be reduced. For example, lard and soy phospholipids could be used instead of high-purity TAG, DOPC, and DOPE. In addition, laboratory preparation methods need to utilize a production line to adapt to the needs of mass production.

## Conclusion and prospects

Since their discovery, LDs have been understood as organelles for storing excess cellular lipids. However, in recent years, intensive studies of LD functions revealed that LDs play essential roles in various biological processes. The functions of LDs go far beyond energy storage and instead play an essential role in lipid and protein processing. It is common for diseases to be associated with LD dysfunction and neutral lipid deficiencies or excesses. It is possible for abnormal LD amounts to disrupt lipid and protein homeostasis, potentially causing disease or at least modulating its severity and outcome. LD traps molecules otherwise harmful to cells or prone to deterioration and destruction. Apolipoproteins, free fatty acids (e.g., free fatty acids), and environmental toxins are examples of the former, while PUFAs and viral components are examples of the latter.

As well as promoting eicosanoid synthesis, protein maturation, folding, and turnover, LDs regulate the metabolism and homeostasis of their cargo, typically lipid or protein. Furthermore, during cellular stress, LDs are essential for maintaining homeostasis, and lipid and protein processing appear to be co-mitigated in the case of ER stress. The discovery of these functions has dramatically enriched the understanding of LDs and highlighted the vital role that LDs play in cellular life activities.

LDs are an ancient and highly conserved organelle whose presence has been observed in organisms ranging from bacteria to higher mammals. The accumulation of LDs is gradually becoming recognized as a prominent feature of various cancers and has attracted increasing attention. The level of LDs was, moreover, identified to be associated with the clinicopathological features and prognosis of cancer, which means that it may be used as a biomarker for cancer diagnosis, recurrence, and survival. Excess intracellular lipids can be stored in LDs to avoid lipotoxicity, which is beneficial for cancer cell survival. Moreover, cancer cells extensively require membrane biogenesis due to their rapid proliferative state. LD also acts as a regulatory hub for energy homeostasis. LDs release fatty acids, which are used for energy production through mitochondrial oxidation and Krebs’ cycle when nutrients are scarce. Thus, during cancer cell invasion, LDs can serve as a source of energy to support the long-distance spread of cells. Notably, the density and motility of LDs correlate with the invasiveness of cancer.

More importantly, accumulating literature suggests that LDs are closely associated with the chemoresistance of cancer cells [107, 108]. Increased LD content leads to impairment of chemotherapy-induced caspase cascade activation and ER stress response, accompanied by a decrease in immune cell death. Therefore, the accumulation of LDs could be developed as a potential predictor of responses to conventional neoadjuvant or immunotherapy in cancer patients with advanced stages of the disease. The LD is a complex functional organelle that plays a central role in many types of cancer, including proliferation, invasion, metastasis, and chemoresistance. The increased storage of lipids in LD facilitates cancer cell survival. Lipotoxicity and endoplasmic reticulum stress can be prevented by storing excess fatty acids and cholesterol in LD. The presence of more LD can increase the availability of lipid substrates and energy for proliferating cancer cells to meet their metabolic demands. There is evidence that LDs provide invasive cancers with energy reserves, which can trigger metastatic cloning in the tumor microenvironment. In addition, the accumulation of LDs may affect drug-induced apoptosis and immune cell death, leading to chemoresistance among cancer cells.

**Fig. 3:**
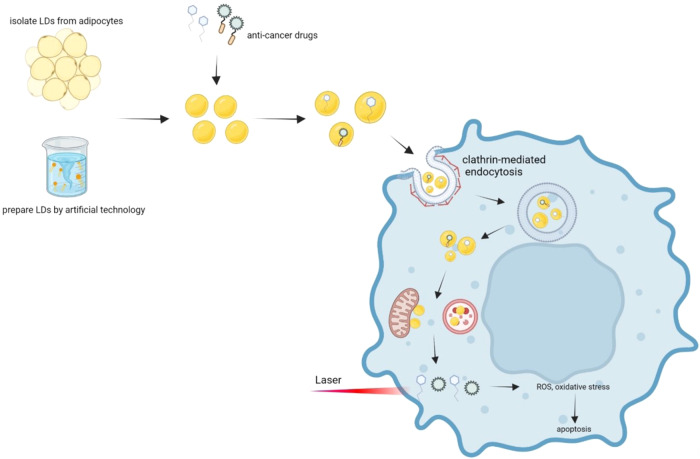
A large number of LDs can be obtained by cellular LD extraction or artificial LD techniques. First, the anticancer drug is encapsulated in the LDs, co-incubating with the cells. The LDs enter cancer cells through lectin-mediated endocytosis, followed by rupturing the endocytic vesicles, and the LDs loaded with drugs are released into the cytoplasm. Then, the LDs can interact with organelles such as lysosomes, mitochondria, and the endoplasmic reticulum. Subsequently, anticancer drugs are released into the cytoplasm. Some of these drugs can directly induce oxidative stress, endoplasmic reticulum stress, and apoptosis. In addition, some photosensitizing drugs function after the laser irradiation of cancer cells to promote apoptosis.

These functions give LDs powerful applications. For example, LDs can aggregate lipid-soluble drugs and lipophilic proteins for anticancer drug delivery and assist in photodynamic therapy to effectively kill cancer cells. Furthermore, LDs can be used as endogenous intracellular microlenses to enhance the performance of multifunctional biocompatible optical tools for biosensing, endoscopic imaging, and single-cell diagnostics to aid biomedical research (Fig. 3). However, because large amounts of LDs are required for drug carriers and cancer therapy, the yield of LDs from extracted cells is low and costly. Nevertheless, accessible and fast methods are already available to prepare large quantities of artificial LDs, which solves the problem of LD yield. In addition, LDs can be made into lyophilized powder for easy storage and use. In summary, as a bionanomaterial, LDs will have critical applications in the biomedical field in the future.
